# Multi-omics analyses demonstrate complement dysregulation is involved in focal segmental glomerulosclerosis

**DOI:** 10.3389/fimmu.2026.1852918

**Published:** 2026-09-09

**Authors:** Yingxue Xu, Sipei Chen, Yong Zhang, Yi Li, Guisen Li

**Affiliations:** 1 Department of Nephrology, Sichuan Provincial People’s Hospital, School of Medicine, University of Electronic Science and Technology of China, Sichuan Clinical Research Center for Kidney Diseases, Chengdu, China; 2 Institutes for Systems Genetics, West China Hospital, Sichuan University, Chengdu, China

**Keywords:** complement, focal segmental glomerulosclerosis, outcome, proteomics, transcriptomic

## Abstract

**Introduction:**

The complement system is increasingly recognized as a contributor to the pathogenesis of focal segmental glomerulosclerosis (FSGS). However, a comprehensive, tissue-based characterization of complement dysregulation in human FSGS kidneys remains lacking.

**Methods:**

We performed an integrative multi-omics analysis combined with histological validation. Proteomic (LC-MS/MS) profiling was conducted on renal cortical tissues from 11 patients with FSGS and 12 healthy controls from our institution, while transcriptomic (RNA-seq) analysis was performed using publicly available datasets comprising 100 FSGS and 30 normal samples. *In situ* deposition of key complement components (C1q, C3c, C5a, C7, CFB, CFH, MBL) was assessed using multiplex immunofluorescence confocal microscopy. Correlations between complement deposition and clinical outcomes were analyzed.

**Results:**

Multi-omics analyses consistently identified the complement and coagulation cascades among the most significantly enriched pathways in FSGS. Transcriptomic data revealed upregulation of C1QB, C1R, C1S, C2, C3, and CFB mRNA, whereas proteomic profiling showed upregulation of C4BPA, C5, C8B, C8G, and CFHR5, along with downregulation of C1R, C1S, C2, CFB, and CFH. In Tmem30a knocked down mouse podocytes, complement pathway enrichment was observed, but core components including C3, C1r, C1s, and CFB were downregulated. Immunofluorescence confirmed frequent glomerular C3c deposition (43.75%), with lower positivity rates for C1q (15.63%), CFB (12.5%), and CFH (12.5%); MBL deposition was rare. Patients with glomerular deposition of C3c, C1q, CFB, or CFH exhibited significantly lower probabilities of achieving complete remission.

**Conclusions:**

This study systematically demonstrates concurrent dysregulation of the classical and alternative complement pathways in FSGS. The deposition of specific complement components in renal tissue strongly correlates with disease activity, treatment resistance, and poor prognosis, suggesting their potential as pathological biomarkers and therapeutic targets. These findings enhance the understanding of FSGS immunopathogenesis and support the strategy of complement-targeted therapies.

## Introduction

Focal segmental glomerulosclerosis (FSGS) represents a common and often progressive pattern of glomerular injury, histologically defined by focal and segmental sclerosis within the glomeruli (1, 2). It is a leading cause of nephrotic syndrome and significantly contributes to the global burden of end-stage kidney disease (ESKD) (3). The pathogenesis of FSGS is multifactorial, traditionally attributed to podocyte injury, genetic predisposition, and hemodynamic stress, leading to its classification as a non-immune complex-mediated glomerulopathy (4, 5).

However, accumulating clinical evidence challenges the notion of FSGS as a observations challenge this purely “non-immune disease” (6). A substantial subset of FSGS kidney biopsy specimens exhibits focal deposition of immunoglobulin M (IgM) and complement component C3 within sclerotic lesions (7). Importantly, the presence and intensity of these deposits are associated with increased disease activity (8) and accelerated decline in renal function (9), suggesting a pathogenic rather than an incidental role for complement activation (10).

There is accumulating multi-level experimental evidence supporting the involvement of complement activation in the pathogenesis of FSGS (11). In an adriamycin-induced FSGS model, the activation of the C3a/C3aR signaling axis on podocytes has been shown to induce actin cytoskeleton rearrangement, directly leading to podocyte injury and progression of glomerulosclerosis (12). Notably, C3-deficient mice exhibit significantly attenuated renal damage in this setting (13). Furthermore, pharmacological blockade of the complement anaphylatoxin receptor C5aR1 effectively reduces proteinuria and ameliorates histopathological injury (14).

Clinical studies further substantiate the pathological relevance of complement activation in FSGS. Renal biopsy analyses demonstrate a significantly higher prevalence of glomerular C1q deposition in FSGS patients compared to those with minimal change disease (MCD) or healthy individuals (15). Moreover, urinary C1q levels are elevated in FSGS relative to membranous nephropathy (MN), IgA nephropathy (IgAN), and lupus nephritis (LN) (16). Intriguingly, plasma C1q levels in FSGS patients are significantly lower than in healthy controls and show a negative correlation with urinary protein excretion (17), suggesting local consumption of C1q at sites of complement activation. Additionally, levels of the alternative pathway activation marker Ba are increased in both plasma and urine of FSGS patients (18, 19). Plasma Ba levels correlate with disease severity (20), while urinary Bb levels are closely associated with therapeutic response and renal outcomes (17, 21). In contrast, activation of the mannose-binding lectin (MBL) pathway is rarely observed in FSGS.

Despite these findings, a systematic, unbiased mapping of the complement system in human FSGS kidney tissue remains lacking. Fundamental questions persist: Which complement activation pathways are predominantly involved? What is the comprehensive expression profile of complement components, regulators, and receptors? Most importantly, does *in situ* complement activation directly influence clinical outcomes such as treatment response and disease recurrence?

To address these critical gaps, we designed an integrated multi-omics and spatial validation study. First, we performed unbiased proteomic and transcriptomic profiling of renal cortices from FSGS patients and controls to delineate the global complement “activation landscape”. Second, we employed multiplex immunofluorescence confocal microscopy to visualize the *in-situ* deposition of key components from all three complement activation pathways. Finally, we rigorously correlated these molecular and histological findings with detailed longitudinal clinical data to evaluate their prognostic and predictive value. This study aims to provide a definitive characterization of complement dysregulation in FSGS and assess its potential as a source of biomarkers and novel therapeutic targets.

## Materials and methods

### Study cohort and ethical approval

This retrospective study utilized renal biopsy tissues and clinical data from 43 patients with primary FSGS and 12 healthy controls (obtained from nephrectomy specimens for non-glomerular diseases) at Sichuan Provincial People’s Hospital between January 2019 and December 2024. The diagnosis of primary FSGS was established by two independent renal pathologists according to the Columbia classification criteria. Secondary FSGS was excluded through clinical evaluation, hemodynamic assessment, and renal imaging studies. Familial and genetic FSGS were excluded by detailed three-generation pedigree analysis and whole-exome sequencing targeting known FSGS-associated pathogenic genes. Primary FSGS was defined as histologically confirmed FSGS in the absence of secondary, genetic, or familial factors. The study protocol was approved by the Institutional Review Board of Sichuan Provincial People’s Hospital, and all procedures adhered to the Declaration of Helsinki.

### Clinical data collection and definitions

Baseline demographic, laboratory, and pathological data were obtained from medical records. Follow-up data included treatment regimens, 24-hour proteinuria, serum creatinine, and estimated glomerular filtration rate (eGFR, calculated using the CKD-EPI equation). Clinical outcomes were defined as follows: Complete Remission (CR): proteinuria <0.5 g/24h with stable renal function (eGFR within ±15% of baseline). Partial Remission (PR): >50% reduction in proteinuria from baseline (or <3 g/24h if initially with nephrotic-range proteinuria) with stable renal function; No Remission (NR): Failure to meet criteria for either CR or PR; Relapse: Recurrence of nephrotic-range proteinuria (>3.5 g/24h) after achievement of remission.

### Proteomic analysis (LC-MS/MS)

Renal cortical tissues from 11 patients with FSGS and 12 controls were homogenized. Proteins were extracted, quantified, and digested with trypsin according to the FASP protocol. Resulting peptides were analyzed by liquid chromatography-tandem mass spectrometry (LC-MS/MS) using a Q Exactive HF-X instrument (Thermo Fisher Scientific). Raw data were processed with MaxQuant software (v.2.0.3) against the UniProt human reference proteome database. Differentially expressed proteins (DEPs) were identified based on |log_2_ (fold change) | > 0.58 and p < 0.05 (Student’s t-test). Functional enrichment analysis of DEPs was conducted using the DAVID bioinformatics platform.

### Transcriptomic data integration and analysis

Publicly available transcriptomic datasets for FSGS (GSE104948, GSE121211, GSE125779, GSE133288, GSE180395) were retrieved from the GEO database. After background correction and normalization using the limma package, batch effects were adjusted using the Combat function from the sva package. Differential expression analysis between 100 FSGS and 30 normal samples was performed with limma, defining differentially expressed genes (DEGs) as those with |log_2_FC| > 0.58 and adjusted p-value (FDR) < 0.05.

### Construction of *Tmem30a* KD mouse podocytes

Tmem30a was knocked down in immortalized mouse podocytes using lentivirus-mediated RNA interference, and phenotypic analysis and functional verification were performed at the cellular level. Cells were transfected for 72 h with the interfering virus, after which Tmem30a knockdown was verified using RT-PCR and western blotting. Differentiated podocytes (control group), transfected negative control lentivirus mouse podocytes (NC group), and *Tmem30a* KD mouse podocytes (*Tmem30a* KD group) were treated with 0.4 μg/mL of ADR for 24h.

### Immunofluorescence confocal microscopy

Formalin-fixed, paraffin-embedded renal sections (2 μm) from 32 FSGS patients were processed for immunofluorescence. After antigen retrieval and blocking with goat serum, sections were incubated overnight at 4 °C with primary antibodies: anti-CFB (Affinity, DF6567, 1:200), anti-CFH (Proteintech, 12748-1-AP, 1:200), anti-MBL (Abcam, ab190834, 1:400), anti-C5a (Abcam, ab281923, 1:200), and anti-C7 (Zen BioScience, 382511, 1:200). Commercially conjugated antibodies were used for C1q (Dako, F0254, FITC, 1:100) and C3c (Dako, F0210, FITC, 1:100). Fluorophore-conjugated secondary antibodies were subsequently applied. Nuclei were counterstained with DAPI. Images were acquired using a Zeiss LSM900 confocal microscope. Staining intensity and distribution were independently evaluated by two pathologists blinded to clinical information. A semiquantitative scale was used to evaluate the intensity of complement deposition. 1+ (weak positive): Faint fluorescence signal requiring careful identification; 2+ (moderate positive): Clear, easily visible fluorescence signal with moderate intensity; 3+ (strong positive): Intense fluorescence signal with extensive coverage. Positive deposition was defined as a semiquantitative score of ≥ 1 +.

### Statistical analysis

Continuous variables are presented as mean ± SD or median (interquartile range) and compared using Student’s t-test or Mann-Whitney U test, as appropriate. Categorical variables are expressed as counts (percentages) and compared using Chi-square or Fisher’s exact test. All statistical tests were two-sided, with significance set at p < 0.05. Analyses were performed using SPSS 21.0 and R software (v.4.5.2).

## Results

### Multi-omics profiling identifies complement and coagulation cascade as a central pathway in FSGS

**Figure 1:**
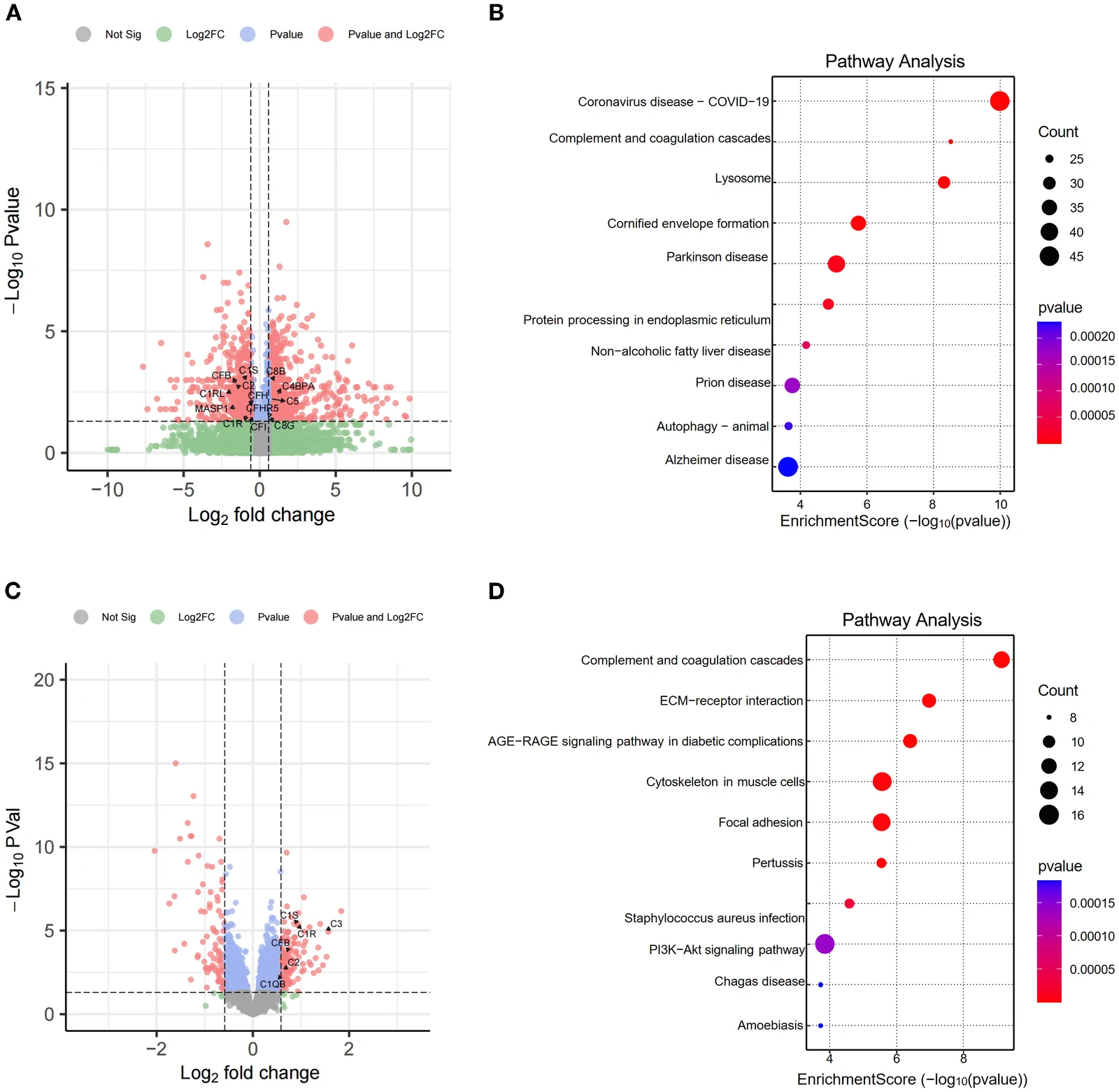
Multi-omics profiling identifies dysregulation of the complement and coagulation cascades in FSGS. **(A)** Volcano plot showing differentially expressed proteins (DEPs) in the renal cortex of FSGS patients (n = 11) compared with healthy controls (n = 12). Red dots represent significantly upregulated and downregulated proteins, respectively (fold change > 1.5, P < 0.05). **(B)** KEGG pathway enrichment analysis of DEPs between FSGS and HC. The Complement and coagulation cascades was identified as the second most significantly enriched pathway. **(C)** Volcano plot showing differentially expressed genes (DEGs) between FSGS patients (n = 100) and healthy controls (n = 30) based on public transcriptomic datasets. Red dots indicate significantly upregulated and downregulated genes, respectively (fold change > 1.5, P < 0.05). **(D)** KEGG pathway enrichment dot plot of DEGs between FSGS and HC. The Complement and coagulation cascades emerged as the most significantly enriched pathway.

The clinical characteristics of the discovery cohort for proteomics are summarized in **Table 1**. Unbiased proteomic analysis of renal cortices from 11 FSGS patients and 12 healthy controls quantified 9,039 proteins, of which 1,349 were differentially expressed (802 upregulated, 547 downregulated; **Figure 1A**). Notably, KEGG pathway enrichment analysis identified the “Complement and Coagulation Cascades” as the second most significantly enriched pathway among these DEPs (**Figure 1B**, **Supplementary Figure 1**). This finding was validated by integrated transcriptomic analysis of 100 FSGS and 30 normal samples from public datasets, in which the same pathway emerged as the top enriched term among the 210 identified DEGs (**Figures 1C, D**, **Supplementary Figure 2**). These convergent multi-omics data strongly suggest systemic dysregulation of the complement system in pathogenesis of FSGS. As shown in **Supplementary Figure 3**, transcriptome analysis of Tmem30a KD podocytes also revealed enrichment of the complement pathway, albeit with a distinct expression pattern (downregulation of C3, C1r, C1s, and CFB, and upregulation of MASP1), suggesting cell-type-specific and context-dependent regulation of complement genes.

**Table 1:** Baseline characteristics of patients with FSGS.

Characteristics	FSGS (n=11)
Age (year)	39.64 ± 5.77^a^
Gender (male, %)	9 (81.8%)
Race (Chinese, %)	11 (100%)
Proteinuria (g/24h)	4.71 ± 1.08
Serum creatinine (μmol/L)	90.80 (93.10)^b^
eGFR (mL/min/1.73 m^2^)	80.83 ± 12.69
Serum albumin (g/L)	29.71 ± 3.54
Serum C3 (g/L)	1.27 ± 0.14
Serum C4 (g/L)	0.36 ± 0.03
Tip Variant n (%)	3 (27.3%)^c^
Not Otherwise Specified n (%)	8 (72.7%)

FSGS, Focal Segmental Glomerulosclerosis.

eGFR, estimated glomerular filtration rate.

^a^Data are expressed as means ± standard deviations.

^b^Data are expressed as medians and interquartile ranges (IQRs).

^c^Data are expressed as frequency and percentage.

### Distinct patterns of complement component dysregulation at transcriptional and protein levels

To further characterize this dysregulation, we focused on 34 complement-related proteins. Proteomic analysis revealed 15 DEPs within this group (**Table 2**). Notably, terminal pathway components (C5, C8B, C8G) and regulator CFHR5 were upregulated, whereas several initiators of the classical and alternative pathways (C1R, C1S, C2) and regulatory proteins (CFB, CFH, CFI) were downregulated. In contrast, transcriptomic data showed significant upregulation of C1R, C1S, C2, C3, and CFB mRNA (**Table 2**). This discordance between reduced protein levels and elevated mRNA expression for key upstream components suggests potential post-transcriptional regulation, increased protein turnover, or local consumption due to intrarenal complement activation in FSGS.

**Table 2:** Differences in complement components at the transcriptional and protein levels.

Complements	Proteomics		Transcriptomics		Complements	Proteomics		Transcriptomics	
	log2FC	P value	log2FC	P value		log2FC	P value	log2FC	P value
C1QA	-0.48	0.26	0.56	9.19×10^-4^	CD46	0.58	0.02	-0.16	1.60×10^-4^
C1QB	-0.75	0.13	0.59	3.63×10^-4^	CD59	0.35	0.53	0.07	0.12
C1R	-0.86	0.03	0.94	2.59×10^-8^	CD55	1.73	0.09	-0.05	0.60
C1QR1	1.18	0.08	0.09	0.33	CFB	-1.55	2.90×10^-4^	0.76	2.69×10^-6^
C1RL	-1.94	3.00×10^-3^	0.45	4.91×10^-6^	CFD	0.25	0.38	0.33	0.01
C1S	-1.09	1.00×10^-3^	0.92	1.64×10^-8^	CFH	-0.73	0.01	0.31	0.04
C2	-1.46	2.90×10^-4^	0.68	1.21×10^-4^	CFHR2	0.42	0.29	0.06	0.46
C3	-0.39	0.65	1.57	7.63×10^-8^	CFHR5	0.62	0.03	-0.12	0.04
C4BPA	1.19	4.00×10^-3^	-0.02	0.77	CFI	-0.63	0.04	0.13	0.13
C4BPB	-0.63	0.57	0.05	0.43	CLU	0.1	0.35	0.63	4.38×10^-6^
C5	0.76	0.01	0.18	0.11	COLEC11	-0.62	0.32	-0.31	0.01
C6	0.35	0.21	0.25	0.13	COLEC12	-3.48	0.15	0.19	0.15
C7	0.46	0.04	0.34	0.01	CR1	0.61	0.35	-0.25	0.10
C8A	0.62	0.35	0.02	0.75	MASP1	-1.71	0.01	0.24	0.02
C8B	0.79	1.00×10^-3^	0.003	0.94	MASP2	-0.69	0.29	-0.08	0.25
C8G	0.74	0.03	0.06	0.30	CPN1	-2.58	4.01×10-^4^	0.04	0.59
C9	0.66	0.26	-0.03	0.73	CPN2	-2.39	1.80×10^-5^	1.13×10^-4^	1.00

### *In situ* validation reveals frequent glomerular deposition of specific complement components

**Figure 2:**
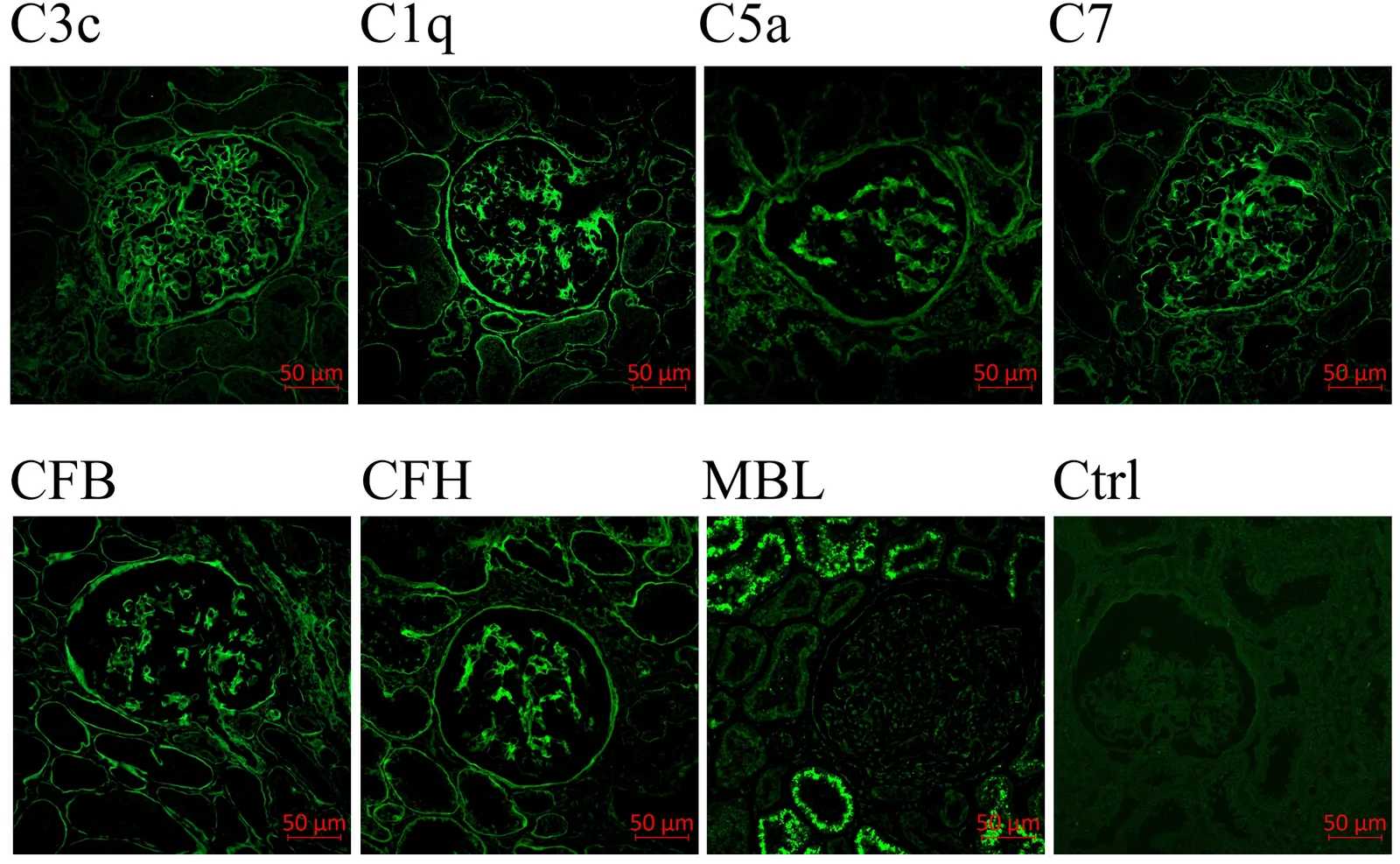
Immunofluorescence detection of complement components in renal biopsies from FSGS patients and control group. Representative immunofluorescence confocal microscopy images showing the deposition of complement components in renal biopsies from FSGS patients and control group. Positive signals for C3c, C1q, C5a, C7, CFB, CFH, and MBL are shown in green. The control exhibited C3c-negative images. Scale bars, 50 μm.

We next examined the patterns of complement deposition in an independent cohort of 32 FSGS patients (characteristics in **Supplementary Table 1**). We found that glomeruli were the predominant sites of complement deposition. Occasional weak complement deposition was observed in the renal tubules, but the intensity was substantially lower than that in glomerular positive cases. Immunofluorescence confocal microscopy revealed that C3c deposition was the most prevalent, detected in 14 patients (43.75%), predominantly along glomerular capillary loops. Deposits of C5a (21.88%), C7 (18.75%), C1q (15.63%), CFB (12.5%), and CFH (12.5%) were less frequent but clearly present. In contrast, MBL deposition was observed in only one patient and was primarily localized to renal tubules, suggesting a minimal role for the lectin pathway (**Figure 2**, **Supplementary Figure 4**).

### Complement deposition correlates with poor treatment response

Of the 32 FSGS patients, 15 (46.9%) achieved complete remission, 10 (31.2%) partial remission, and 7 (21.9%) no remission. Among the patients who achieved remission, 13 (46.4%) experienced subsequent relapse. There were no significant differences in serum creatinine or proteinuria levels between patients with and without C3c deposition. Importantly, the presence of complement deposits was associated with significant clinical implications. Patients with C3c deposition achieved complete remission at a significantly lower rate (21.4%)compared to those without C3c deposition (**Table 3**). Patients with C3c deposition showed a numerically higher relapse rate, although this difference did not reach statistical significance (*p* = 0.149). Similarly, positivity for C1q, CFB, or CFH was associated with low CR rates and, while positivity for C7, CFB, or CFH was linked to higher relapse rates (**Supplementary Table 2**). Multivariable logistic regression was performed to assess the association between C3c deposition and complete remission. After adjusting for baseline eGFR and proteinuria and tubular atrophy/interstitial fibrosis, C3c deposition remained associated with lower odds of complete remission (OR = 0.061, 95% CI 0.004-0.980, *p* = 0.048). Given the limited number of outcome events relative to the number of covariates, these results should be interpreted with caution. C3c deposition is not definitive evidence of an independent predictor of treatment response.

**Table 3:** Comparison of clinical characteristics and treatment responses between C3c-positive and C3c-negative patients.

Characteristics	C3c (+)n=14	C3c (-)n=18	P
Age (year)	31.43 ± 10.86^a^	39.28 ± 17.04	0.144
Proteinuria (g/24h)	5.91 (2.83)^b^	8.63 (6.16)	0.070
Serum creatinine (μmol/L)	82.8 (87.8)	70.3 (56.45)	0.210
eGFR (mL/min/1.73 m^2^)	95.73 (81.46)	100.84 (56.91)	0.691
Serum albumin (g/L)	22.64 ± 6.79	18.56 ± 4.64	0.053
Serum C3 (g/L)	0.92 (0.35)	1.07 (0.39)	0.289
Serum C4 (g/L)	0.29 ± 0.11	0.32 ± 0.08	0.397
Tubular atrophy/interstitial fibrosis n (%)	5 (35.7%)^c^	6 (33.3%)	0.777
Monocyte infiltration n (%)	5 (35.7%)	3 (16.7%)	0.224
Treatment Regimens			0.722
Glucocorticoid monotherapy	5 (35.7%)	7 (38.9%)	
Glucocorticoid + Calcineurin inhibitor	8 (57.1%)	8 (44.4%)	
Glucocorticoid + Cyclophosphamide	1 (7.1%)	2 (11.1%)	
Rituximab	0 (0%)	1 (5.6%)	
Complete Remission n (%)	3 (21.4%)	12 (66.7%)	0.012
Partial Remission n (%)	7 (50%)	3 (16.7%)	0.047
No Remission n (%)	4 (28.6%)	3 (16.7%)	0.426
Relapse n (%)	7 (63.6%)	6 (35.3%)	0.149

eGFR, estimated glomerular filtration rate.

^a^Data are expressed as means ± standard deviations.

^b^Data are expressed as medians and interquartile ranges (IQRs).

^c^Data are expressed as frequency and percentage.

## Discussion

This study presents the first integrated multi-omics characterization of complement system dysregulation in human FSGS. Our principal findings are threefold: (1) unbiased proteomic and transcriptomic analyses converge on the complement and coagulation cascade as a central pathogenic pathway; (2) there is evidence of concurrent dysregulation of both classical (C1q) and alternative (CFB, CFH) pathway at the protein deposition level, despite complex discrepancies between transcript and protein expression; (3) critically, *in situ* deposition of specific complement components, particularly C3c, serves as a robust histopathological biomarker associated with treatment resistance and disease recurrence. We demonstrate complement component deposition in FSGS glomeruli, we acknowledge that deposition alone does not conclusively establish active cascade engagement. Established activation markers such as C4d, Bb and C5b-9/MAC were not assessed due to limited tissue availability and antibody accessibility. Our detection of C5a and C3c provides partial evidence of terminal pathway activation, but future studies with dedicated activation-specific antibodies are needed to definitively confirm ongoing complement activation in FSGS.

The high prevalence of C3c deposits (43.8%) is consistent with previous pathological reports and highlights their central role in disease pathogenesis. Our clinical correlation data significantly extend these findings by mechanistically linking C3c deposition to adverse outcomes. These observations support the hypothesis that sustained complement activation—likely mediated through C3a/C5a anaphylatoxin signaling on podocytes and inflammatory cells—drives persistent glomerular injury and sclerosis (12, 14), contributing to reduced responsiveness to standard immunosuppressive therapies (22). This provides a strong rationale for evaluating C3 or C5 inhibitors in selected patients with FSGS (23).

Complement deposition in FSGS may be secondary to passive IgM accumulation in segmental sclerotic areas. IgM, a macroglobulin, can become trapped in the sclerotic extracellular matrix due to size-selective retention or increased affinity for the matrix, where it may subsequently activate the classical pathway via C1q binding. Distinguishing between primary (immune-mediated) versus secondary (sclerosis-associated) complement activation in human biopsy specimens is challenging, and both mechanisms may co-exist.

A novel and seemingly paradoxical finding were the downregulation of several upstream complement proteins (C1R, C1S, CFB, CFH) alongside upregulated corresponding mRNAs. We propose that this is not contradictory, but rather reflects active protein consumption. In the context of chronic, low-grade complement activation, these proteins may undergo continuous cleavage and tissue depletion, prompting a compensatory increase in gene transcription. This pattern mirrors is consistent with observations in other complement-mediated disorders and further supports the concept of an “activated” state within the FSGS glomeruli (24, 25).

Notably, the complement expression profiles in human renal tissues and the podocyte-specific Tmem30a knockout model exhibited marked discrepancies in this study. While the mRNA levels of C3, C1r, and C1s were upregulated in the renal cortex of patients with FSGS, these same core complement components were consistently downregulated in Tmem30a KD mouse podocytes. We speculate that the elevated complement transcripts observed in human kidney tissues may primarily originate from infiltrating immune cells such as macrophages, or from activated resident glomerular cells including endothelial cells. In contrast, podocytes themselves may downregulate the synthesis of key complement components like C3 and CFB as a compensatory protective mechanism to reduce *in situ* complement complex formation and deposition. These findings suggest that complement dysregulation in FSGS involves a complex network of multiple cell types and multi-level regulation, in which podocytes serve not only as targets of complement attack but also as active participants in disease progression through the modulation of their own complement expression profile.

Our analysis indicates that the complement dysregulation in FSGS primarily occurs via the classical and alternative pathways. The coexistence of both C1q and CFB/CFH deposits suggests that neither the classical nor the alternative pathway acts independently, and their concurrent engagement may contribute to the phenotypic heterogeneity observed in FSGS. The near absence of MBL deposition suggests that the lectin pathway, if involved, does not play a predominant role in this cohort. However, we acknowledge that MBL deposition alone is an incomplete marker of lectin pathway activation, and a more comprehensive assessment is required to definitively rule out the possibility of lectin pathway activation. Importantly, the clinical correlations of these deposits underscore their pathological significance: they are not incidental findings but markers of a more aggressive disease phenotype 6. Thus, immunofluorescence detection of complement components may serve as a valuable adjunct in prognostic renal pathological assessment.

Several limitations should be acknowledged. The retrospective, descriptive nature of this study precludes definitive mechanistic conclusions. Our findings establish robust associations between complement dysregulation and clinical outcomes in FSGS, but cannot distinguish whether complement activation is a primary driver of disease versus a secondary consequence of podocyte injury or non-specific trapping in sclerotic lesions. Mechanistic validation will require prospective studies with longitudinal sampling, animal models with cell-specific complement deficiencies, or therapeutic trials of complement inhibitors. This study is limited by its small sample size. For instance, the limited number of positive cases in the immunofluorescence validation cohort reduced the statistical power of the prognostic analyses. Notably, the observed difference in relapse rates between C3c-positive and C3c-negative patients (63.6% vs. 35.3%, *p* = 0.149) did not reach statistical significance, which may reflect insufficient statistical power rather than the absence of a true effect. Therefore, validation in larger cohorts with adequate statistical power is required. The proteomic and transcriptomic analyses were performed on distinct patient cohorts. Proteomics utilized data from 11 FSGS patients from our institution, whereas transcriptomics leveraged publicly available datasets. Potential confounding may be introduced due to cohort heterogeneity, including differences in disease severity, treatment, and population characteristics. Future studies using paired multi-omics data from the same patients will be needed to definitively resolve the discrepancies between transcriptomic and proteomic data.

## Data Availability

The datasets presented in this study can be found in online repositories. The names of the repository/repositories and accession number(s) can be found below: http://www.proteomexchange.org/, PXD083528.

## References

[1] D’Agati VD Kaskel FJ Falk RJ . Focal segmental glomerulosclerosis. N Engl J Med. (2011) 365:2398–411. doi: 10.1056/NEJMra1106556 22187987

[2] Altintas MM Agarwal S Sudhini Y Zhu K Wei C Reiser J . Pathogenesis of focal segmental glomerulosclerosis and related disorders. Annu Rev Pathol. (2025) 20:329–53. doi: 10.1146/annurev-pathol-051220-092001 39854184 PMC11875227

[3] Rosenberg AZ Kopp JB . Focal segmental glomerulosclerosis. Clin J Am Soc Nephrol. (2017) 12:502–17. doi: 10.2215/CJN.05960616 28242845 PMC5338705

[4] Wiggins RC . The spectrum of podocytopathies: a unifying view of glomerular diseases. Kidney Int. (2007) 71:1205–14. doi: 10.1038/sj.ki.5002222 17410103

[5] Fogo AB . Causes and pathogenesis of focal segmental glomerulosclerosis. Nat Rev Nephrol. (2015) 11:76–87. doi: 10.1038/nrneph.2014.216 25447132 PMC4772430

[6] Salfi G Casiraghi F Remuzzi G . Current understanding of the molecular mechanisms of circulating permeability factor in focal segmental glomerulosclerosis. Front Immunol. (2023) 14:1247606. doi: 10.3389/fimmu.2023.1247606 37795085 PMC10546017

[7] Strassheim D Renner B Panzer S Fuquay R Kulik L Ljubanović D . IgM contributes to glomerular injury in FSGS. J Am Soc Nephrol. (2013) 24:393–406. doi: 10.1681/ASN.2012020187 23393315 PMC3582199

[8] Zhang Y-M Gu Q-H Huang J Qu Z Wang X Meng L-Q . Clinical significance of IgM and C3 glomerular deposition in primary focal segmental glomerulosclerosis. Clin J Am Soc Nephrol. (2016) 11:1582–9. doi: 10.2215/CJN.01190216 27340287 PMC5012474

[9] Mirioglu S Caliskan Y Ozluk Y Dirim AB Istemihan Z Akyildiz A . Co-deposition of IgM and C3 may indicate unfavorable renal outcomes in adult patients with primary focal segmental glomerulosclerosis. Kidney Blood Press Res. (2019) 44:961–72. doi: 10.1159/000501827 31437846

[10] Caliskan Y Royal V Troyanov S Bonnefoy A Merlen C Schnitzler M . Clinical significance of immune deposits and complement system activation in FSGS: Findings from the Cure Glomerulonephropathy Network Study. Kidney360. (2025) 6:1384–93. doi: 10.34067/KID.0000000787 40168593 PMC12407132

[11] Lenderink AM Liegel K Ljubanović D Coleman KE Gilkeson GS Holers VM . The alternative pathway of complement is activated in the glomeruli and tubulointerstitium of mice with adriamycin nephropathy. Am J Physiol Renal Physiol. (2007) 293:F555–564. doi: 10.1152/ajprenal.00403.2006 17522263

[12] Angeletti A Cantarelli C Petrosyan A Andrighetto S Budge K D’Agati VD . Loss of decay-accelerating factor triggers podocyte injury and glomerulosclerosis. J Exp Med. (2020) 217:e20191699. doi: 10.1084/jem.20191699 32717081 PMC7478737

[13] Turnberg D Lewis M Moss J Xu Y Botto M Cook HT . Complement activation contributes to both glomerular and tubulointerstitial damage in adriamycin nephropathy in mice. J Immunol. (2006) 177:4094–102. doi: 10.4049/jimmunol.177.6.4094 16951374

[14] Gong X-J Huang J Shu Y Wang M Ji J Yang L . Complement C5a and C5a receptor 1 mediates glomerular damage in focal segmental glomerulosclerosis. Clin Immunol. (2025) 273:110459. doi: 10.1016/j.clim.2025.110459 39984108

[15] van de Lest NA Zandbergen M Wolterbeek R Kreutz R Trouw LA Dorresteijn EM . Glomerular C4d deposition can precede the development of focal segmental glomerulosclerosis. Kidney Int. (2019) 96:738–49. doi: 10.1016/j.kint.2019.04.028 31327475

[16] Genest DS Bonnefoy A Khalili M Merlen C Genest G Lapeyraque A-L . Comparison of complement pathway activation in autoimmune glomerulonephritis. Kidney Int Rep. (2022) 7:1027–36. doi: 10.1016/j.ekir.2022.02.002 35571000 PMC9091805

[17] Huang J Cui Z Gu Q-H Zhang Y-M Qu Z Wang X . Complement activation profile of patients with primary focal segmental glomerulosclerosis. PloS One. (2020) 15:e0234934. doi: 10.1371/journal.pone.0234934 32569286 PMC7307932

[18] Chebotareva N Vinogradov A Tsoy L Varshavskiy V Stoljarevich E Bugrova A . CD44 expression in renal tissue is associated with an increase in urinary levels of complement components in chronic glomerulopathies. Int J Mol Sci. (2023) 24:7190. doi: 10.3390/ijms24087190 37108355 PMC10138917

[19] Morita Y Ikeguchi H Nakamura J Hotta N Yuzawa Y Matsuo S . Complement activation products in the urine from proteinuric patients. J Am Soc Nephrol. (2000) 11:700–7. doi: 10.1681/ASN.V114700 10752529

[20] Thurman JM Wong M Renner B Frazer-Abel A Giclas PC Joy MS . Complement activation in patients with focal segmental glomerulosclerosis. PloS One. (2015) 10:e0136558. doi: 10.1371/journal.pone.0136558 26335102 PMC4559462

[21] Chebotareva NV Vinogradov A Brzhozovskiy AG Kashirina DN Indeykina MI Bugrova AE . Potential urine proteomic biomarkers for focal segmental glomerulosclerosis and minimal change disease. Int J Mol Sci. (2022) 23:12607. doi: 10.3390/ijms232012607 36293475 PMC9604469

[22] Hodson EM Sinha A Cooper TE . Interventions for focal segmental glomerulosclerosis in adults. Cochrane Database Syst Rev. (2022) 2:CD003233. doi: 10.1002/14651858.CD003233.pub3 35224732 PMC8883337

[23] Wada T Nangaku M . Novel roles of complement in renal diseases and their therapeutic consequences. Kidney Int. (2013) 84:441–50. doi: 10.1038/ki.2013.134 23615508

[24] Petr V Thurman JM . The role of complement in kidney disease. Nat Rev Nephrol. (2023) 19:771–87. doi: 10.1038/s41581-023-00766-1 37735215

[25] Portilla D Sabapathy V Chauss D . Role of local complement activation in kidney fibrosis and repair. J Clin Invest. (2025) 135:e188345. doi: 10.1172/JCI188345 40519169 PMC12165786

