# Supplementary material for: Multi-omics analyses demonstrate complement dysregulation is involved in focal segmental glomerulosclerosis
Source: Front Immunol. 2026 Sep 9;17:1852918. doi: 10.3389/fimmu.2026.1852918 (PMC13597383; doi:10.3389/fimmu.2026.1852918)

Supplementary Table S1. Baseline characteristics of 32 patients with FSGS.

**Characteristics**	**FSGS (n=32）**
Age (year)	32.50 (13.5)[b]
Proteinuria (g/24h)	7.46 (6.75)
Serum creatinine (μmol/L)	78.45 (56.47)
eGFR (mL/min/1.73 m^2)	100.84 (61.04)
Serum albumin (g/L)	20.35 ± 5.95[a]
Serum C3 (g/L)	1.01 (0.35)
Serum C4 (g/L)	0.31 ± 0.10
Tip Variant n (%)	9 (28.125%)[c]
Cellular Variant n (%)	1 (3.125%)
Not Otherwise Specified n (%)	22 (68.75%)

a Data are expressed as means ± standard deviations.

b Data are expressed as medians and interquartile ranges (IQRs).

c Data are expressed as frequency and percentage.

Supplementary Table S2. Association between complement components and outcome.

**Outcome**	**C5a (+)**	**C5a (-)**	**C7 (+)**	**C7 (-)**	**C1q (+)**	**C1q (-)**	**CFB (+)**	**CFB (-)**	**CFH (+)**	**CFH (-)**
Relapse n (%)	3 (50.0%)	10 (45.4%)	4 (80.0%)	9 (39.1%)	2 (50.0%)	11 (45.8%)	3 (100.0%)	10 (40.0%)	3 (100.0%)	10 (40.0%)
No Remission n (%)	1 (14.3%)	6 (24.0%)	1 (16.7%)	6 (23.1%)	1 (20.0%)	6 (22.2%)	3 (75.0%)	4 (14.3%)	2 (50.0%)	5 (17.9%)
Partial Remission n (%)	3 (42.3%)	7 (28.0%)	3 (50.0%)	7 (26.9%)	3 (60.0%)	7 (26.0%)	1 (25.0%)	9 (32.1%)	2 (50.0%)	8 (28.6%)
Complete Remission n (%)	3 (42.3%)	12 (48.0%)	2 (33.3%)	13 (50.0%)	1 (20.0%)	14 (51.9%)	0 (0.0%)	15 (53.5%)	0 (0.0%)	15 (53.5%)

Supplementary Figure S1. Bioinformatics analysis in the focal segmental glomerulosclerosis (FSGS) and healthy controls (HC). (A) GO enrichment diagram of differentially expressed proteins between FSGS and healthy controls. (B) GO enrichment diagram of differentially expressed genes between FSGS and healthy controls.

A

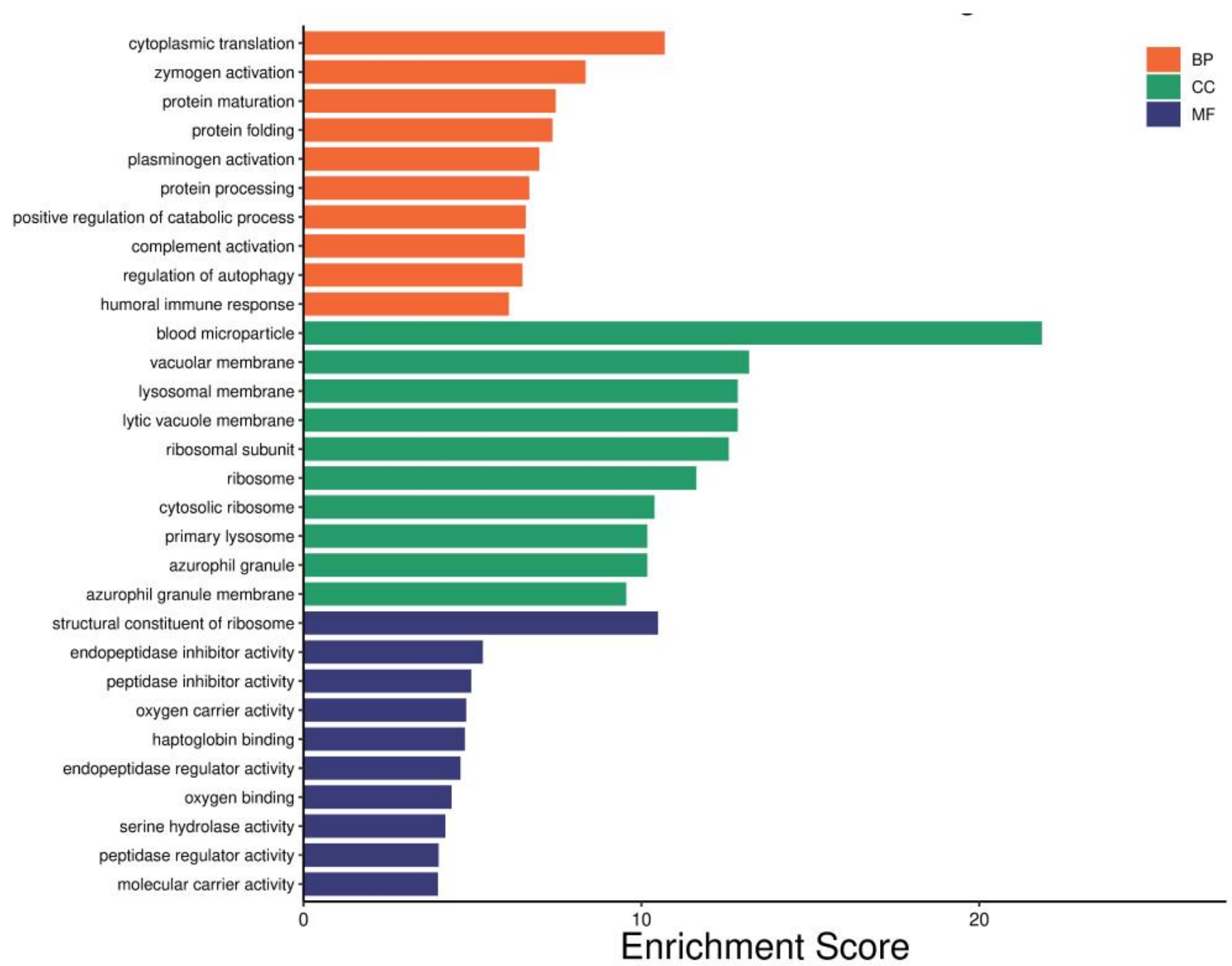

B

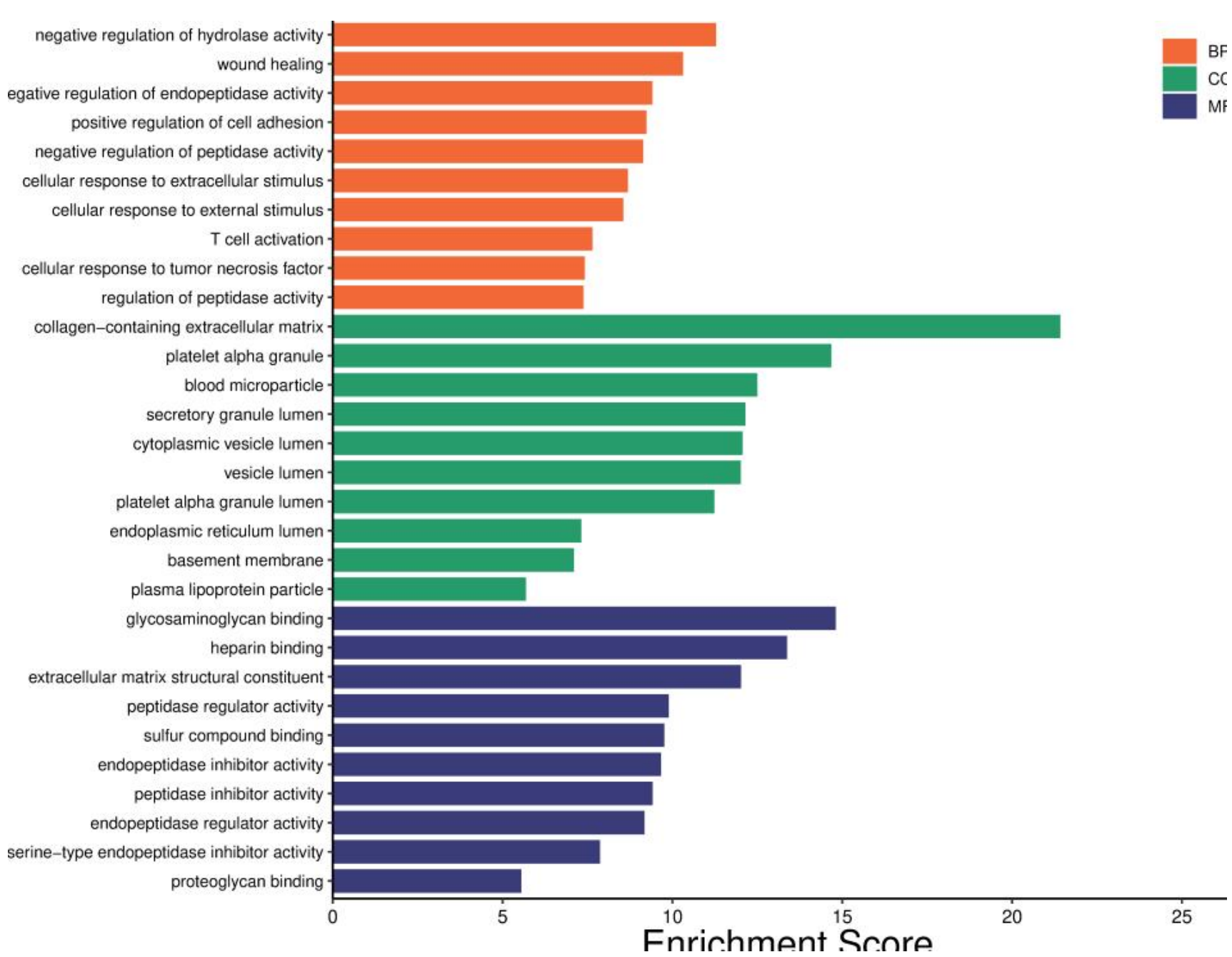

Supplementary Figure S2. Transcriptomics data merging and batch correction. (A)Before batch correction. (B)After batch correction.

A

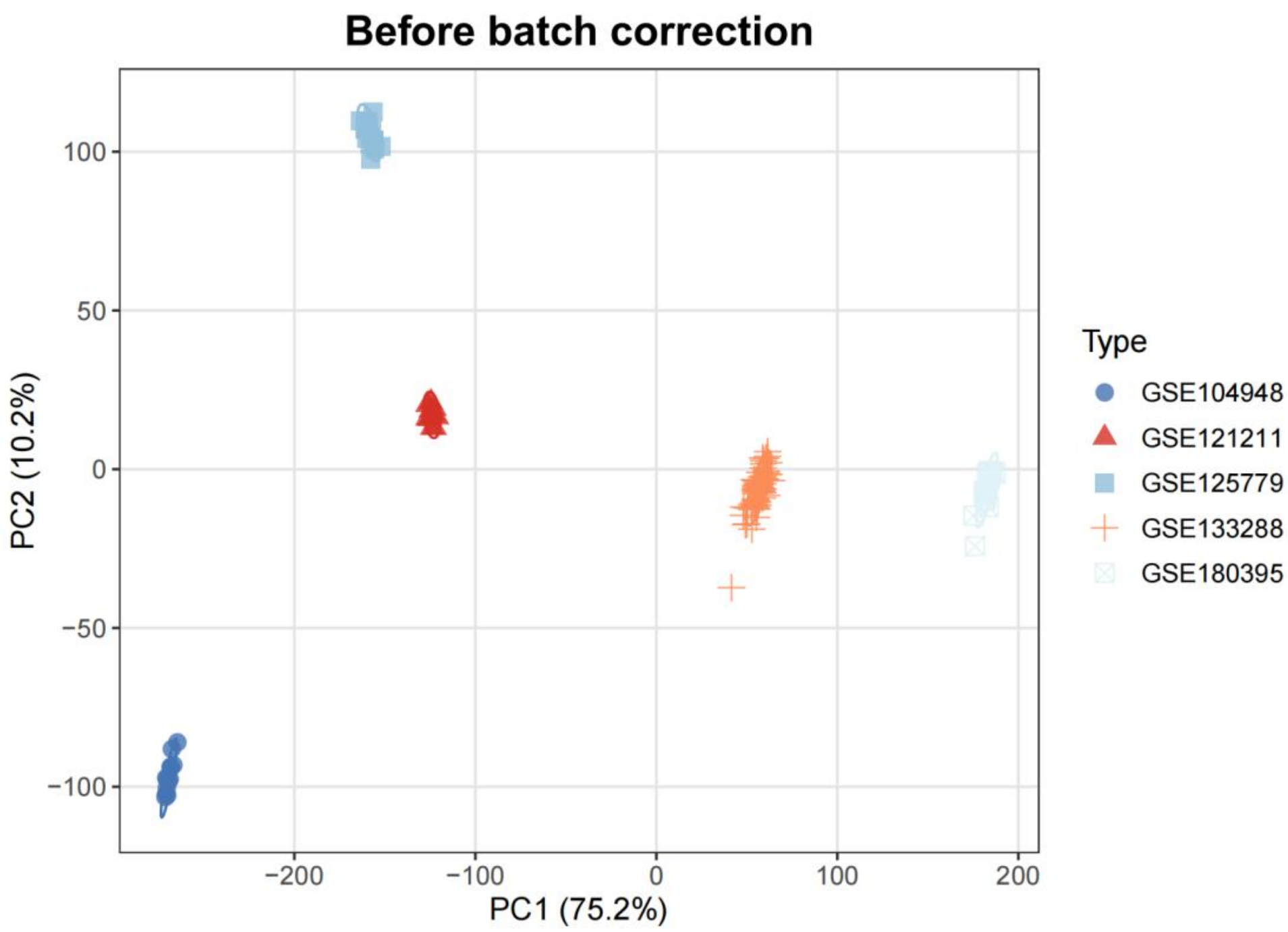

B

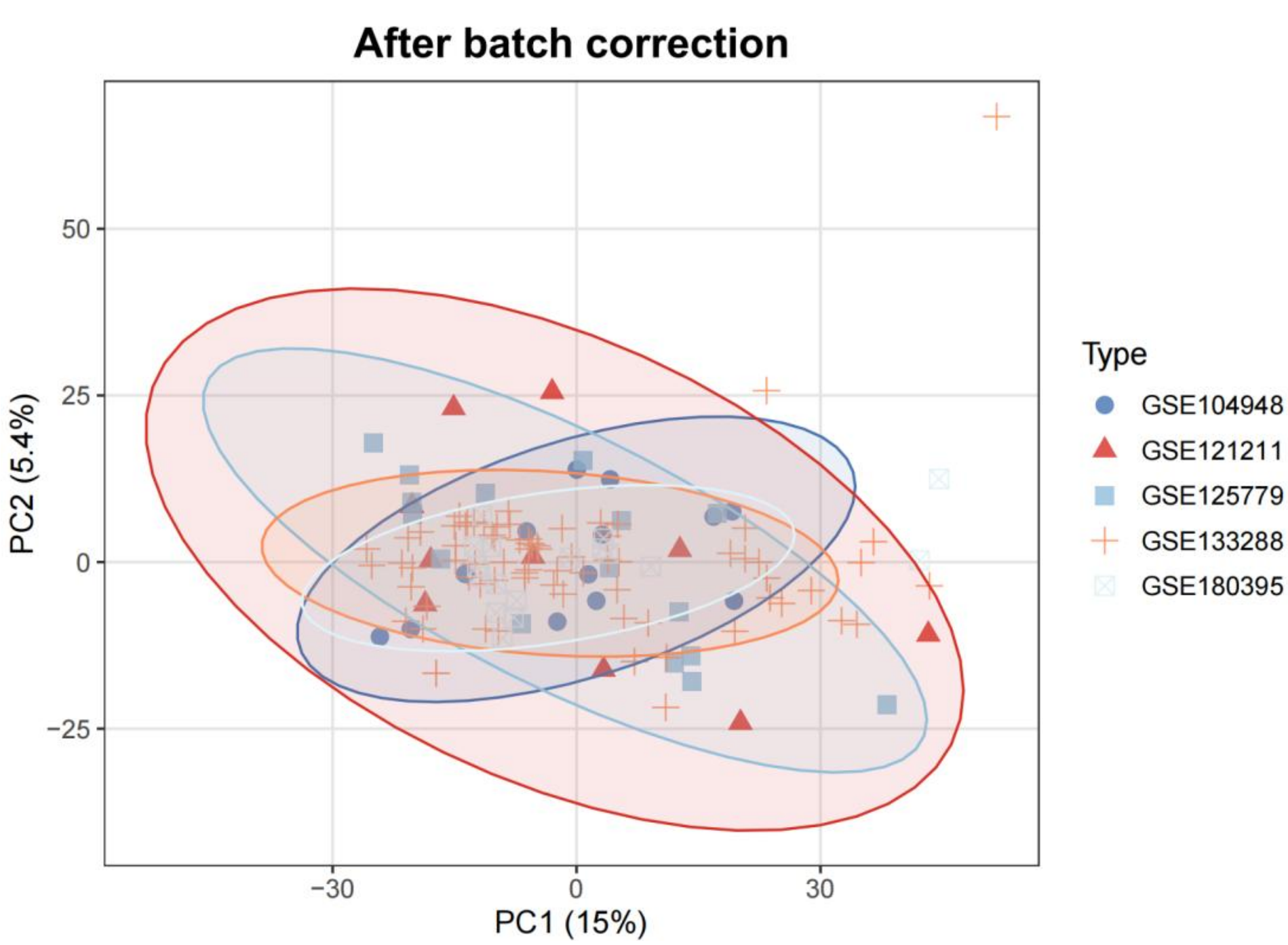

Supplementary Figure S3. Transcriptome sequencing of Tmem30a-knockout podocytes reveals significant enrichment of the complement pathway.

(A) Volcano plot showing differentially expressed genes (DEGs) in Tmem30a KD mouse podocytes compared with wild-type (WT) controls. Red and blue dots represent significantly upregulated and downregulated genes, respectively ($|\text{fold change}| > 2$, $P<0.05$). Gray dots indicate genes with no significant difference.

(B) KEGG pathway enrichment dot plot of DEGs between Tmem30a KD and WT mouse podocytes. The size of each dot represents the number of genes enriched in the corresponding pathway. The Complement and coagulation cascades was identified as a significantly enriched pathway.

(C) Heatmap showing expression changes of complement-related genes in Tmem30a KD mouse podocytes compared with WT controls. Color scale represents row-scaled expression levels (red: upregulated; blue: downregulated). Core complement components, including C3, C1r, C1s, and CFB were significantly downregulated in Tmem30a KD mouse podocytes, whereas MASP1 expression was markedly upregulated.

A

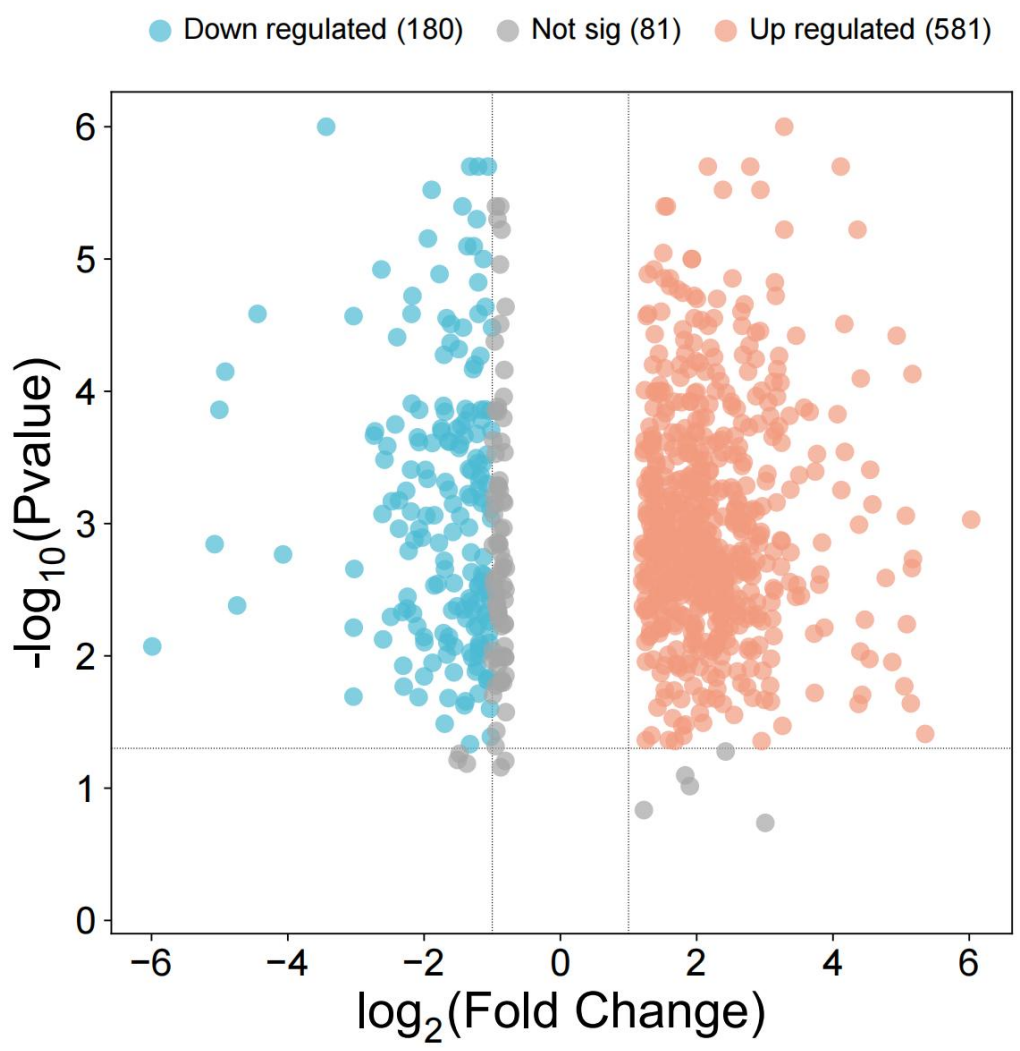
Down regulated (180)
Not sig (81)
Up regulated (581)
-log10(Pvalue)
log2(Fold Change)

B

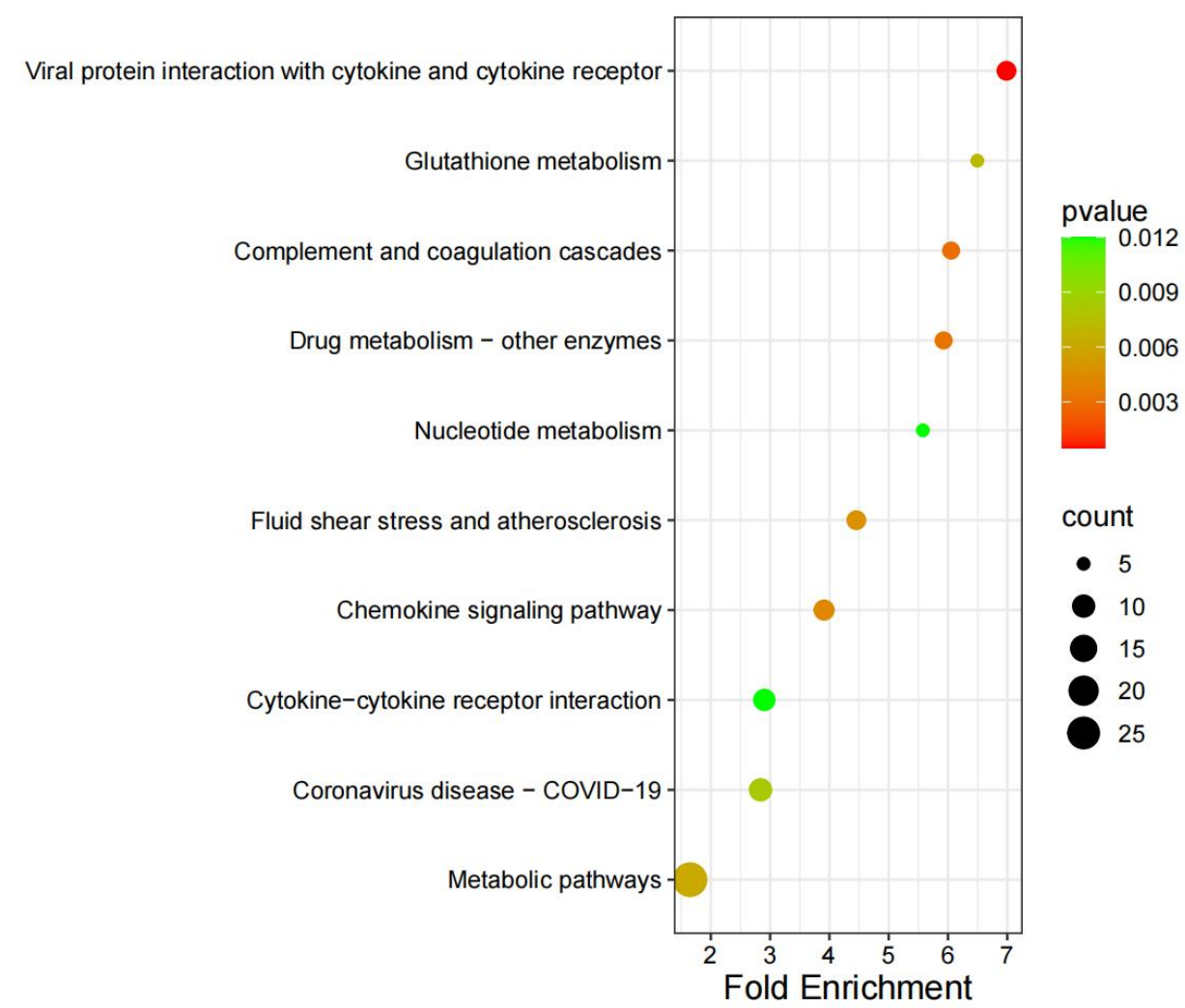
Viral protein interaction with cytokine and cytokine receptor
Glutathione metabolism
Complement and coagulation cascades
Drug metabolism − other enzymes
Nucleotide metabolism
Fluid shear stress and atherosclerosis
Chemokine signaling pathway
Cytokine−cytokine receptor interaction
Coronavirus disease − COVID−19
Metabolic pathways
Fold Enrichment
pvalue
count

C

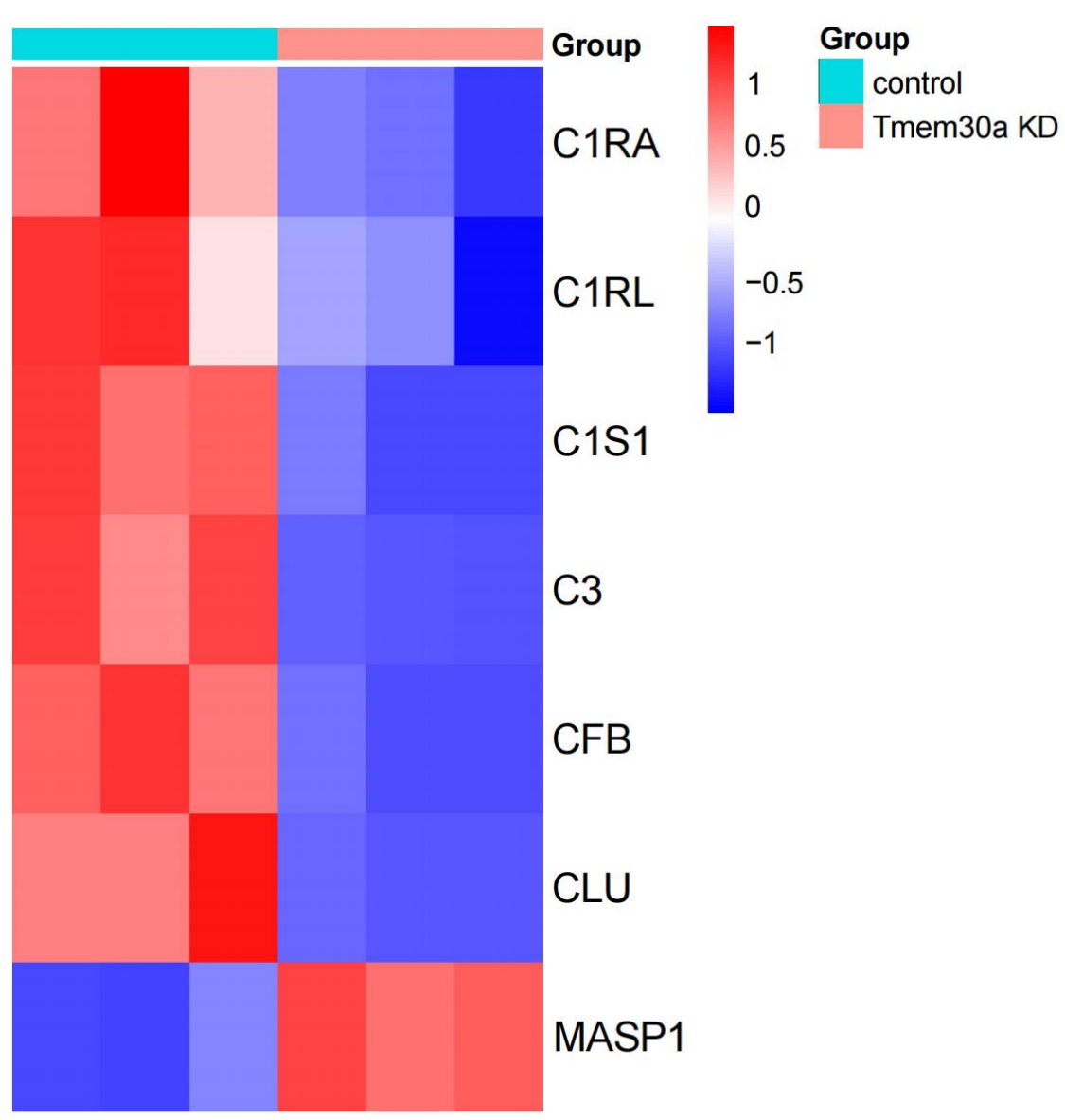
Group
C1RA
C1RL
C1S1
C3
CFB
CLU
MASP1
Group
control
Tmem30a KD

Supplementary Figure S4. Complete immunofluorescence staining results showing positive signals for C3c, C1q, C5, C7, CFB, CFH, and MBL.

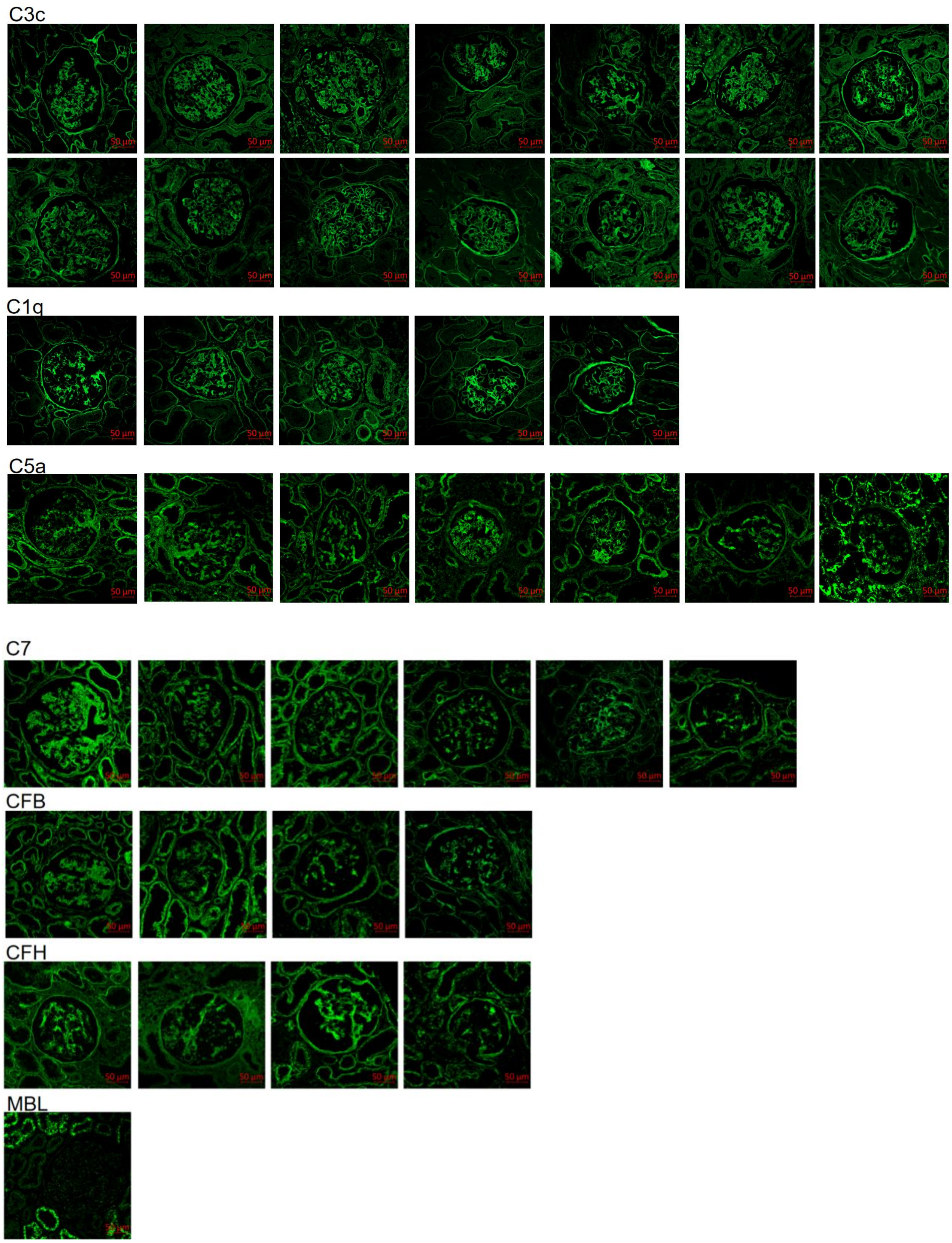

Supplement: Supplementary file 1 [file DataSheet1.pdf]
